# The 2026 FIFA World Cup: Beyond risk, towards epidemiological opportunity

**DOI:** 10.1016/j.nmni.2026.101795

**Published:** 2026-06-16

**Authors:** Giancarlo Ceccarelli, Francesco Branda, Fabio Scarpa, Massimo Ciccozzi

**Affiliations:** a Department of Public Health and Infectious Diseases, University of Rome Sapienza, Rome, Italy; b Azienda Ospedaliero Universitaria Umberto I, Rome, Italy; c Genomics, AI, Bioinformatics, Infectious Diseases, Epidemiology Group (GABIE), Rome, Italy; d Migrant and Global Health Research Organization (Mi-HeRO), Rome, Italy; e Unit of Medical Statistics and Molecular Epidemiology, Università Campus Bio-Medico di Roma, Rome, Italy; f Department of Biomedical Sciences, University of Sassari, Sassari, Italy

**Keywords:** FIFA World Cup 2026, Mass gatherings, Epidemiological infrastructure, Population health, Human mobility

Dear Editor,

As preparations intensify for the 2026 FIFA World Cup, discussions surrounding infectious disease risks have understandably focused on outbreak preparedness, cross-border surveillance, international travel, and the potential importation of communicable diseases. Such concerns are justified. The tournament will involve an unprecedented scale of mobility, bringing together 48 national teams across 16 host cities distributed throughout the United States, Canada, and Mexico. However, framing the World Cup primarily as a public health threat may overlook a far more important scientific opportunity [1,2].

Mass gathering medicine has traditionally approached large events through the lens of risk mitigation. The central question has often been how to prevent outbreaks associated with crowd concentration within stadiums, fan zones, transportation hubs, and host cities. This framework has proved invaluable for protecting public health during international sporting and religious gatherings [1]. Yet the 2026 FIFA World Cup differs fundamentally from most previous mass gatherings.

Rather than a single event or a collection of independent local gatherings, the tournament will temporarily create a continent-wide system of interconnected urban, transportation, healthcare, environmental, and social infrastructures operating simultaneously across North America. Millions of spectators, workers, volunteers, media personnel, and support staff will move repeatedly between geographically distant metropolitan areas over a relatively short period. Consequently, the defining epidemiological characteristic of the tournament may not be crowd density alone, but connectivity [3,4].

This conceptual transition from an event-centred perspective to an infrastructure-centred perspective is illustrated in Fig. 1 (not shown). The framework proposes the tournament as a temporary epidemiological infrastructure generated by the interaction of human mobility, urban systems, environmental interfaces, healthcare networks, wastewater systems, and digital connectivity across multiple countries [1,5].

**Fig. 1:**
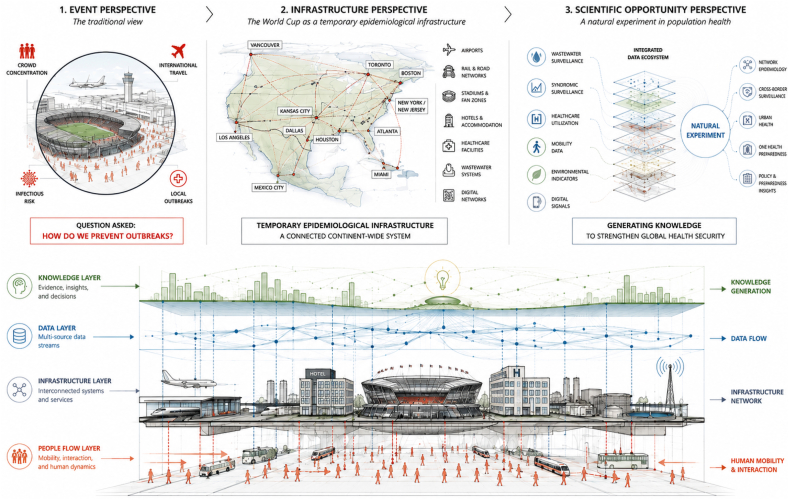
**The 2026 FIFA World Cup as a temporary epidemiological infrastructure.** Conceptual representation of the transition from an event centred view of mass gatherings to an infrastructure centred perspective. Human mobility, interconnected urban systems, environmental interfaces, healthcare networks, wastewater systems, and digital surveillance platforms collectively generate a temporary epidemiological observatory capable of supporting large scale population health research and preparedness activities beyond outbreak prevention alone. The framework illustrates how interconnected infrastructures may simultaneously shape transmission opportunities and generate observational data streams that can contribute to network epidemiology, cross border surveillance, urban health research, and One Health preparedness.

From this point of view, the World Cup may be more appropriately understood as a temporary epidemiological infrastructure: a large-scale, time-limited socio-spatial system capable of reshaping opportunities for pathogen transmission through the reorganisation of human movement, interaction, and exposure. Under such a framework, epidemiological processes emerge not only within stadiums or host cities but also across the dynamic networks connecting them [3,4].

Importantly, this interpretation extends beyond infectious disease risk. Temporary epidemiological infrastructures may also function as unique scientific observatories. Rarely do researchers have the opportunity to observe, within a defined temporal window, the interactions between large-scale human mobility, urban systems, environmental conditions, healthcare utilisation, wastewater monitoring, and disease surveillance across multiple countries operating within a shared organisational framework [2,5].

The 2026 FIFA World Cup may therefore represent one of the largest natural experiments in population health ever assembled. The tournament will connect diverse climatic, ecological, and urban environments ranging from temperate Canadian cities to subtropical and tropical regions of the United States and Mexico. It will simultaneously link airports, rail systems, hotels, stadiums, healthcare facilities, wastewater networks, digital communication platforms, and public health surveillance systems into a single functional ecosystem driven by predictable and measurable population flows [[2], [3], [4], [5]].

Such conditions create an unprecedented opportunity to investigate how epidemiological signals emerge, propagate, and interact across interconnected environments. Integrated analyses combining syndromic surveillance, wastewater monitoring, environmental indicators, transportation flows, healthcare utilisation patterns, and digital mobility data could generate insights into transmission dynamics that remain difficult to capture under routine conditions [[3], [4], [5]]. The tournament may also provide a unique platform for evaluating novel approaches to cross-border surveillance, network epidemiology, urban health monitoring, and One Health preparedness at continental scale [2].

This perspective invites a broader reconsideration of how mass gatherings are conceptualised within public health. Historically, preparedness has focused on preventing adverse events occurring within a specific venue or locality. However, future epidemics will increasingly emerge within systems characterised by mobility, interdependence, infrastructural complexity, and human–environmental interfaces. Understanding how these systems function may be as important as responding to the threats they generate [3,4].

Astronomers do not value eclipses because they disrupt the sky, but because they reveal phenomena that are otherwise difficult to observe. Likewise, the scientific importance of the 2026 FIFA World Cup may lie not in the infectious disease threats it could generate, but in its capacity to illuminate relationships between mobility, infrastructure, environment, and health that often remain hidden during everyday life.

The legacy of the tournament may therefore extend well beyond sport and beyond preparedness itself. Rather than viewing the World Cup exclusively as a challenge to be managed, it may be more productive to recognise it as a temporary epidemiological infrastructure and a continental-scale observational platform capable of generating knowledge about the interactions between mobility, infrastructure, environment, and health. In doing so, the event could help transform how public health conceptualises future mass gatherings, not merely as settings of potential risk, but as opportunities to generate knowledge essential for understanding the interconnected world in which future epidemics will emerge.

## CRediT authorship contribution statement

**Giancarlo Ceccarelli:** Conceptualization, Investigation, Writing – original draft, Writing – review & editing. **Francesco Branda:** Investigation, Writing – original draft, Writing – review & editing. **Fabio Scarpa:** Investigation, Writing – original draft, Writing – review & editing. **Massimo Ciccozzi:** Supervision, Validation, Writing – original draft, Writing – review & editing.

## Declaration of competing interest

The authors declare that they have no known competing financial interests or personal relationships that could have appeared to influence the work reported in this paper.
